# Combining Radiation and Immune Checkpoint Blockade in the Treatment of Head and Neck Squamous Cell Carcinoma

**DOI:** 10.3389/fonc.2019.00122

**Published:** 2019-03-06

**Authors:** Gregor Manukian, Voichita Bar-Ad, Bo Lu, Athanassios Argiris, Jennifer M. Johnson

**Affiliations:** ^1^Department of Radiation Oncology, Thomas Jefferson University, Philadelphia, PA, United States; ^2^Department of Medical Oncology, Thomas Jefferson University, Philadelphia, PA, United States

**Keywords:** radiation therapy, immunotherapy, immune checkpoint inhibitors, PD1, PD-L1, abscopal effect

## Abstract

Head and neck squamous cell carcinoma (HNSCC) is a significant cause of morbidity and mortality worldwide. Current treatment options, even though potentially curative, have many limitations including a high rate of complications. Over the past few years immune checkpoint inhibitors (ICI) targeting cytotoxic lymphocyte antigen-4 (CTLA-4), programmed cell death protein 1 (PD-1), and programmed cell death ligand 1 (PD-L1) have changed treatment paradigms in many malignancies and are currently under investigation in HNSCC as well. Despite improvements in treatment outcomes and the implementation of combined modality approaches long-term survival rates in patients with locally advanced HNSCC remain suboptimal. Accumulating evidence suggests that under certain conditions, radiation may be delivered in conjunction with ICI to augment efficacy. In this review, we will discuss the immune modulating mechanisms of ICI and radiation, how changing the dose, fractionation, and field of radiation may alter the tumor microenvironment (TME), and how these two treatment modalities may work in concert to generate durable treatment responses against HNSCC.

## Introduction

Head and neck cancer is the sixth most common cancer worldwide, with ~600,000 newly diagnosed cases and 350,000 deaths annually (1). The vast majority of these cancers are squamous cell carcinomas. Most patients with HNSCC present with locally advanced disease and are usually managed with combined modality therapy often incorporating radiation therapy (RT) and chemotherapy. Despite this, ~50% of patients with high-risk disease experience disease recurrence within 3 years of follow up (2, 3). Those who do develop a recurrence have limited treatment options that are often associated with significant morbidity and poor prognosis, emphasizing the need for alternative treatment options (4).

It is now well-accepted that the immune system plays an important role in preventing tumor development and progression. Our growing understanding of adaptive immune responses has led to the discovery of various checkpoints that are often exploited by cancer to evade immune mediated destruction. Immune checkpoint inhibitors have therefore been developed with the goal of overcoming this form of immune-evasion and are currently in clinical use for various disease sites including those of the head and neck. Indeed, numerous clinical trials have demonstrated improvement in overall survival (OS) and progression free survival (PFS) with the use of these agents in both the metastatic and locally-advanced disease setting. Unfortunately, only 20–30% of patients typically respond to treatment, and even fewer have responses that persist beyond 6 months (5).

Radiation therapy is a fundamental modality in the treatment of HNSCC. While the immune modulating properties of RT were first reported in the 1970s (6), harnessing this affect to faithfully produce meaningful clinical responses has proven difficult. Recent case reports describing systemic disease responses after combined RT and ICI however has led to the hypothesis that combined therapy may work synergistically to improve treatment outcomes (7, 8).

The goal of this review is thus to discuss the roles of combined ICI and radiation in the treatment of HNSCC. First, we performed a thorough literature search to include peer reviewed preclinical studies and reviews that highlight the current understanding of the immune system's role in tumor development and the importance of checkpoints in curtailing the immune response. Next, we discuss the tumoricidal effects of radiation, how it modulates the immune response, and how dose, fractionation, and field size can potentially affect treatment outcomes. Lastly, we examine the findings of various clinical trials registered on www.clinicaltrials.gov and that have either been published in peer reviewed journals or presented at societal meetings, that investigate combined therapy and their implications for the future management of HNSCC.

## Immune Checkpoint Inhibition and its Role in Tumor Immunity

Initially proposed by Paul Ehrlich over 100 years ago and formally defined by Burnet and Thomas some 50 years later, it is now accepted that the immune system actively protects the host from neoplastic processes, a phenomenon known as cancer immunoediting (9, 10). A full discussion of this hypothesis is reviewed in detail elsewhere (11–15).

Suffice it to say that cluster of differentiation (CD)8^+^ cytotoxic T lymphocytes (CTLs) are instrumental to the immunoediting process. These cells have evolved to detect intracellular antigens, including those from viral pathogens, which are displayed on the cell surface by major histocompatability (MHC) class I molecules. Antigenic peptides are recognized by the T cell receptor (TCR) which is specific for a single antigen. Engagement of the TCR by the peptide-MHC class I complex triggers T cell mediated apoptosis of the target cell via release of cytotoxic granules containing perforin and granzymes, release of cytokines such as interferon (IFN)-γ and tumor necrosis factor (TNF)-α, and direct interactions via Fas-Fas ligand (16, 17).

Given the highly destructive nature of CTLs, their activation and activity are tightly regulated via so-called immune-checkpoints. They first require activation, or priming, by antigen presenting cells (APC)s which consists of three signals and typically occurs in draining lymph nodes (DLN). Signal one is engagement of the TCR with the peptide-MHC class I complex on the surface of the APC. Signal 2 occurs through binding of the co-stimulatory molecules CD80/CD86 (also known as B7-1 and B7-2, respectively) by the APC with CD28 expressed by the T cell. Signal 3 occurs when interleukin (IL)-2 binds to CD25 on the T cell in an autocrine fashion promoting progression through the cell cycle (18, 19).

Modulation of the immune response can occur at signal 2 through the competitive binding of CD28 by CTLA-4, also known as CD152. CTLA-4 has a 500–2,500-fold higher binding affinity compared with CD80/86 and results in decreased IL-2 production, decreased CTL proliferation, and arrest of T cell activation (20). CTLA-4 blockade improves antitumor immunity by shifting the balance back toward immune activation (21). Ipilimumab, a monoclonal antibody that inhibits CTLA-4, has demonstrated improvements in PFS, OS, response rates, and response duration in patients with either metastatic or locally advanced melanoma in two separate Phase III clinical randomized trials and has demonstrated activity in multiple other disease types (22, 23).

Once activated the CTL will circulate in the periphery, searching for any cell expressing its cognate antigen. Recognition of antigen will result in T-cell directed apoptosis as described above. The target cell however can once again evade destruction through the expression of PD-L1. PD-L1 is additionally expressed by monocytes, regulatory T cells (Tregs), B cells, dendritic cells, and other tumor infiltrating lymphocytes. Engagement of PD-L1 with its receptor, PD-1, expressed by CTLs upon activation, triggers an intracellular cascade that interferes with TCR/CD28 signaling. This in turn results in decreased cytokine production and inhibits cell cycle progression. Chronic exposure to PD-1 signaling generates T cell exhaustion and tolerance even in the face of “actionable antigens” (24, 25). While constitutive expression of PD-L1 by healthy cells prevents unintended injury to surrounding bystander cells, its exploitation by cancers, such as melanoma and HNSCC, contributes to evasion of immune-mediated killing. Monoclonal antibodies targeting either PD-1 (nivolumab, pembrolizumab, cemiplimab) or PD-L1 (atezolizumab, avelumab, durvalumab) have therefore been developed to overcome this mechanism of resistance. In early clinical trials, several of these agents have demonstrated efficacy in various disease sites including colorectal cancer, non-small cell lung cancer, melanoma, renal cell carcinoma and will be discussed in greater detail below.

## The Mechanism of Action of Radiation Therapy

### Radiation as a Therapeutic Modality

Radiotherapy is the use of high energy electromagnetic waves (X-rays or γ-rays), charged particles (electrons, protons, or alpha particles), or other modalities to treat both malignant and benign diseases (26). Absorption of ionizing radiation, measured in Gray (Gy), by biologic tissue causes deoxyribonucleic acid (DNA) strand breaks, either directly or indirectly via the generation of reactive oxygen species (ROS), resulting in cell death via autophagy, necrosis, or apoptosis. In order to minimize normal-tissue toxicity, the total dose of radiation needed to achieve tumor kill is often “fractionated” into smaller doses, typically delivered in a daily fashion (26). While variable depending on the tumor type, location, or presence of gross disease, doses of 50–70 Gy are delivered in 1.5–2.25 Gy per fraction for cancers of the head and neck.

Technological advancements in the delivery of external beam radiation therapy including CT-based inverse planning, multi-leaf collimation, patient immobilization, and active image guidance, have led to the development of techniques such as stereotactic body radiation therapy (SBRT) and stereotactic radiosurgery (SRS) which allow for the precise delivery of very high doses of radiation in 1 to 5 fractions. These techniques are currently in use for the treatment of brain and bone metastases, early stage non-small cell lung cancer, pancreatic cancer, prostate cancer, and recurrent head and neck cancers (27–30). While these high doses of radiation result in irreversible lethal DNA damage, both preclinical and clinical data now suggest that changes in the TME may also contribute to tumor control (31).

### How RT Promotes an Anti-tumor Immune Response

The tumoricidal effects of RT appear to at least in part be dependent on an intact immune system. In 1979, Slone et al. demonstrated that thymectomized mice required twice the dose of radiation to achieve cure compared with mice with intact immune systems (6). Effects of RT on the immune response have been seen in antigen presentation, effector T cell recruitment, creation of an immunosuppressive tumor microenvironment, and the expression of immune checkpoint receptors.

#### The Importance of Adjuvant Signaling

**Figure 1:**
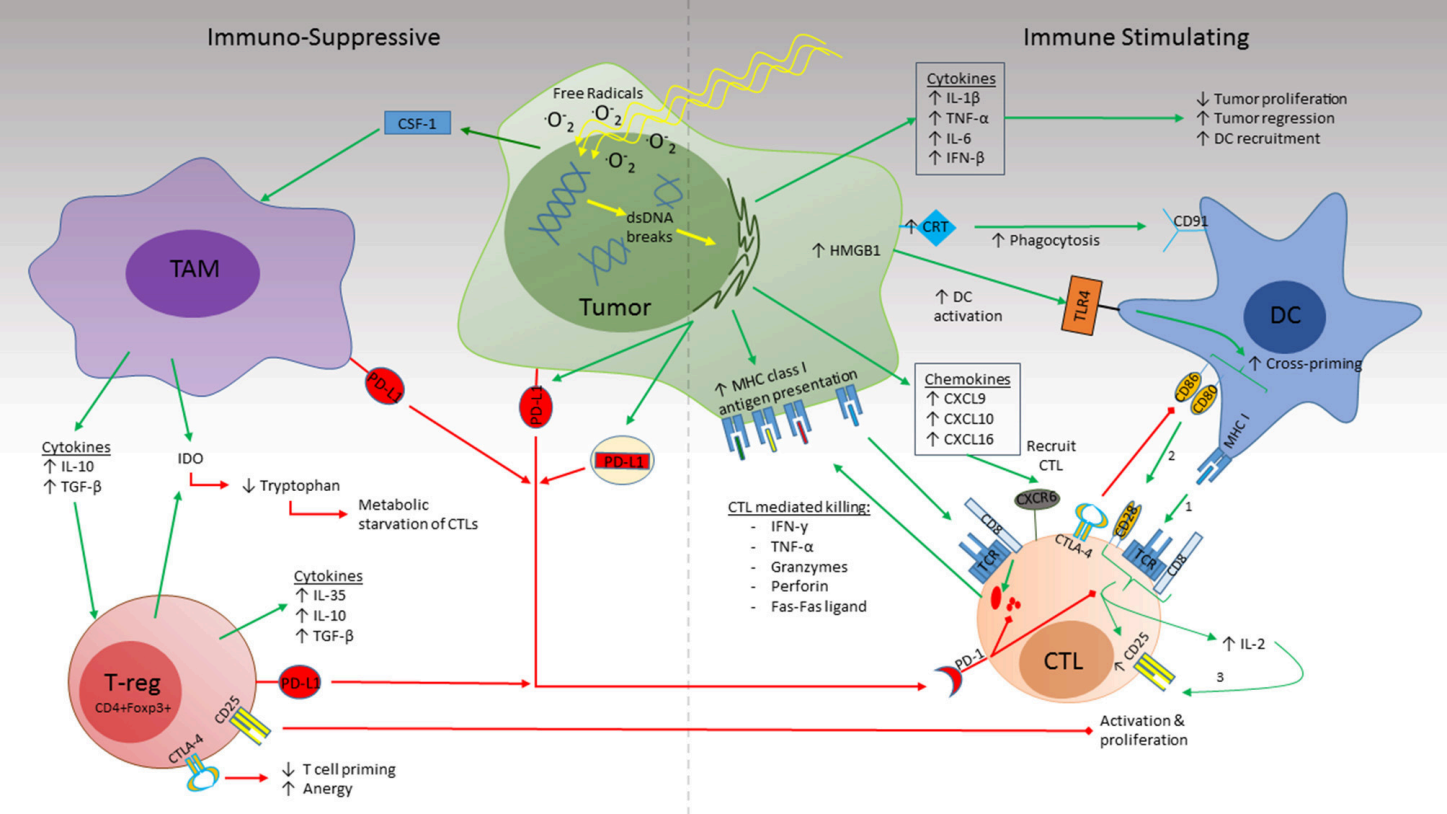
Radiation simultaneously induces immune suppression and immune activation in the TME. RT induces dsDNA damage both directly and indirectly by ROS formation. Activation (**Right**): RT triggers release of the cytokines IL-1β, TNF-α, and IL-6 which promote inflammation and inhibit tumor proliferation while IFN-β promotes DC recruitment. CRT expression by the irradiated tissue binds to CD91 on DCs which promotes phagocytosis. Increased antigen uptake by DCs and activation by HMGB1 binding to TLR4 leads to CTL cross priming in draining lymph nodes. Recognition of cognate antigen by the naïve CTL provides Signal 1 required for CTL maturation. Co-stimulation (signal 2) by CD80/86 on the DC with CD28 on the CTL leads to upregulation of the high-affinity IL-2 receptor, CD25, as well as IL-2 secretion by the CTL which promotes T cell proliferation and survival. Release of the chemokines CXCL9, CXCL10, and CXCL16 recruit activated CTLs to the TME which recognize their cognate antigen via MHC class I molecules on the tumor surface. This in turn initiates cytolysis via release of cytokines (IFN-γ, TNF-α), cytotoxic granules (Granzymes, perforins), and direct cell-cell interactions (Fas-Fas ligand). Suppression (**Left**): CSF-1 promotes recruitment of TAMs to the TME. Production of IL-10 and TGF-β by the TAM promotes Treg recruitment. Together, release of IDO enhances tryptophan consumption resulting in CTL starvation. PD-L1 expression by tumor, TAMs, and Tregs impairs cytotoxicity of activated CTL and promotes exhaustion. Expression of CD25 by Tregs competes with CTL uptake of IL-2 thus indirectly impairing CTL proliferation and survival. CTLA4 expressed by both Tregs and DCs competes with CD80/CD86 for CD28 co-stimulation (signal 2) thus preventing CTL activation and promoting anergy.

Similar to how T cells require multiple signals for successful priming, dying cells need to express both exogenous or mutated antigens as well as adjuvant signals in order to elicit an antigen specific immune response. This may in part explain why cells undergoing accidental necrosis, such as that from freeze thawing or osmotic shock, fail to generate protective immunity (32–35). The adjuvant signals in question come in the form of damage-associated molecular pattern (DAMP)s such as adenosine triphosphate (ATP), high mobility group protein 1 (HMGB1), and calreticulin (CRT), which bind to their respective pattern recognition receptors (PRR)s. After RT, CRT is upregulated by irradiated tumor cells which acts as a pro-phagocytic signal via CD91 on activated APCs. Meanwhile, HMGB1, which is also elevated after RT, binds to TLR4 receptors on dendritic cells (DC) resulting in increased activation. These activated APCs begin taking up antigen and promote CTL cross-priming as discussed above (Figure 1) (35–38).

Chemotherapies, such as paclitaxel and oxaliplatin, have also been shown to promote immunogenic cell death (ICD) via CRT, ATP, HMGB1, and various heat shock proteins (33). Combined with radiation, Golden et al. demonstrated that platinums and taxanes increase the pro-immunogenic repertoire from dying tumor cells that could facilitate host anticancer immune responses (36). Interestingly cisplatin (CDDP), an alkylating agent commonly used concurrently with RT in treating HNSCC and is in the same drug class as oxaliplatin, fails to induce ICD. This is likely due to cisplatin's inability to trigger CRT translocation from the lumen to the endoplasmic reticulum (ER), a process which is dependent on the phosphorylation of eukaryotic translation initiation factor 2α (eIF2α), the formation of ER stress, and initiation of macroautophagy. The authors however demonstrate that tumor immunogenicity with CDDP is possible through the addition of an ER stress inducer such as tunicamycin (39).

#### The Importance of Antigenicity

As mentioned earlier, antigenicity, in the form of neo-antigens, in combination with strong adjuvant signals is required to generate a robust adaptive immune response. This has been observed in human malignancies with a high mutational burden due to mismatch repair deficiency. Specifically, patients with mismatch repair deficient colorectal cancers who were treated with pembrolizumab experienced a statistically significant improvement in immune-related progression free survival of 78% compared with 11% in those whose tumors were mismatch repair-proficient (40).

Tumors with a *low* mutational burden however may become antigen rich through the addition of radiation. Reits et al. demonstrated that RT induced the expression of unique proteins involved in DNA repair, cell cycle check-points, apoptosis, and protein degradation, that were subsequently loaded and presented by host MHC class I molecules to effector T cells (41). Similarly, a study by Garnett et al. assessing the responses of 23 human cancer cell lines after non-lytic doses of radiation found that 91% up-regulated one or more surface molecules involved in CTL mediated killing (42).

Of course, immune responses can be provoked against foreign antigens such as viral DNA. As a large subset of HNSCC stem from either human papilloma virus (HPV) or Epstein Barr virus (EBV) infections, these types of antigens may play an important role in immune stimulation. Thus taken together, these studies suggest that radiation may act as an *in situ* vaccine (43).

Once activated CTLs depend on recognition of their cognate antigen presented via MHC class I molecules on the host cell to initiate cell killing. One method used by malignant cells to evade CTL mediated killing is by downregulating and impairing MHC class I peptide presentation (44, 45). Radiation however upregulates MHC expression in various human cancer cell lines (46–48). This process however may be dose dependent as MHC class I expression in a melanoma cell line increased over 2-fold at doses of ionizing radiation of 10–25 Gy but not at doses of 1 or 4 Gy (41).

#### Radiation Triggers Increased Cytokine and Chemokine Secretion

Radiation also leads to an increased release of cytokines and chemokines which promotes T cell trafficking and priming (49). This is initiated through the detection of DNA damage by cyclic guanosine monophosphate (GMP)-adenosine monophosphate (AMP) synthase (cGAS). The binding of non-sequence specific DNA to cGAS triggers the synthesis of cyclin GMP-AMP (cGAMP) which in turn acts as a messenger that binds to the ER-membrane adaptor stimulator of interferon genes (STING). Through a series of phosphorylation reactions, STING ultimately leads to the activation of the transcription factors interferon regulatory factor 3 (IRF3) and nuclear factor-kB (NF-kB) (50, 51). These transcription factors then travel to the nucleus where they induce the expression of type 1 interferons, IL-1β, IL-6, and TNF-α up to 6 h after radiation (52, 53) (Figure 1). Of these cytokines, the type 1 interferon, IFN-β, is critical in producing the antitumor immunity of RT; type 1 IFN knockout mice exhibited abrogated T cell priming compared with their wild-type controls (54). Furthermore, STING deficient mice fail to reject tumor after local radiation highlighting the importance of the cGAS-STING signaling pathway in RT tumor immunity (55).

Ionizing radiation also upregulates chemokines such as CXC-motif chemokine 9 (CXCL9), and CXCL10, which are involved in the recruitment of activated CD8^+^ T cells (56). CXCL16, which recruits CXCR6 expressing Th1 and CD8^+^ effector T cells, is upregulated by both mouse and human breast cancer cells; CXCR6 deficient mice experienced impaired tumor regression and decreased CD8^+^ T cell infiltration after irradiation (57, 58). IFN-γ produced after RT has also been shown to enhance MHC class I expression and CTL trafficking (38, 59).

### How RT May Suppress the Anti-tumor Immune Response

Like a double-edged sword radiation can also create an immunosuppressive environment through the recruitment of tumor associated macrophages (TAMs), myeloid derived suppressor cells (MDSCs), and CD3^+^CD4^+^CD25^+^Foxp3^+^ Tregs (Figure 1). TAM recruitment is dependent on colony stimulating factor (CSF)-1 which is increased after radiation. Once present, TAMs secrete IL-10 and transforming growth factor-β (TGF-β) which inhibits DC maturation and promotes Treg activation, induce T cell anergy via PD-L1 expression, and create metabolic starvation by expression of indoleamine-pyrrole 2,3-dioxygenase (IDO) (60, 61). Meanwhile, Tregs promote immunosuppression by consumption of IL-2 which is necessary for CTL activation, secretion of IL-10, TGF-β, and IL-35, expression of IDO, and upregulation of CTLA-4 which competes with CD28 binding of B7.1 and B7.2 necessary for T cell priming by APCs (38, 62, 63).

Preclinical models also suggest that Tregs may be radioresistant compared to their CTL counterparts. Using the murine TRAMP C1 model of prostate cancer in mice treated with and without RT, Kachikuwu et al. demonstrated an increased number of Tregs after both local and whole body radiation. In fact these cells persisted in the spleen after doses as high as 20 Gy and maintained their suppressive potential *in vitro* (64, 65). Furthermore, Schaue et al. demonstrated that Treg recruitment may be based on radiation dose. Using a mouse model of melanoma treated with varying doses of radiation revealed that a dose of 15 Gy resulted in a higher proportion of regulatory T cells compared with 5 Gy (66). Fractionation did not appear to have significant effect on Tregs however.

Dovedi et al. showed that radiation at dose of 10 Gy in 5 fractions resulted in upregulation of PD-L1 expression in mouse models of melanoma, colorectal cancer, and triple negative breast cancer. Expression changes were detected as early as 1 day after RT, peaking at 72 h before returning to baseline levels at day 7. This phenomenon was dependent on CD8^+^ T cell production of IFN-γ (67). RT also has been shown to increase PD-1 expression on CD8^+^ and CD4^+^ T cells (68). In humans, PD-L1 expression was significantly increased in patients with previous concurrent chemoradiotherapy for locally advanced esophageal cancer. This was correlated with poorer OS compared to patients with lower PD-L1 levels (69).

## The Potential Importance of Radiation Dose and Fractionation

In 2009, Lee et al. demonstrated that in a B16 melanoma mouse model, a single ablative dose of 20 Gy led to tumor regression that corresponded to an increase in infiltrating T cells to the TME and lymphoid tissue. In the same study, using a metastatic breast cancer model, single fraction ablative radiation (between 15 and 25 Gy) led to complete resolution of distant lung metastases. The response however was abrogated when the radiation was fractionated, specifically 20 Gy in 4 fractions, over 2 weeks (70). These findings were partially confirmed by Schaue et al. in a study where B16-OVA mice were treated with either 5, 7.5, 10, or 15 Gy delivered in a single fraction. Tumor regression was observed at doses higher than 5 Gy. In contrast to the findings by Lee, fractionating the dose into either 5, 3, or 2 fractions had superior tumor responses (66).

Vanpouille-Box et al. offer a mechanism that may explain this response. Using the TSA mouse breast cancer model, they demonstrated that one or three 8 Gy doses of radiation increases the production of double stranded DNA compared with either 20 or 30 Gy single fraction doses. At these higher single fraction doses, an elevation in the exonuclease, Trex1, which plays an essential role in clearing cytoplasmic DNA, was detected. Knocking down Trex1 expression abrogated the abscopal response. The threshold for Trex1 upregulation ranged from 12 to 18 Gy across various mouse and human carcinoma cell lines. Additionally, increasing amounts of cytoplasmic dsDNA triggered the release of IFN-β, which is involved in DC recruitment. This was significantly increased in the 8 Gy times 3 regimen vs. any of the single fraction schemes and was critical in eliciting anti-tumor T cell responses (71).

## Combining Radiation and Immunotherapy

To determine whether immune checkpoint blockade can enhance the response to radiation, Demaria et al. utilized the 4T1 mouse mammary model and treated mice with either a monoclonal antibody against CTLA-4 (9H10) alone, RT (24 Gy in 1 or 2 fractions) to the primary tumor alone, or RT in combination with 9H10. Anti-CTLA4 therapy alone did not delay tumor growth or improve survival whereas RT alone delayed growth of the primary lesion. Combination therapy significantly improved OS and resulted in fewer lung metastases. Depletion of CD8^+^ and CD4^+^ T cells confirmed that this process was depended on the presence of CD8^+^ T cells (72).

PD-1 blockade similarly enhances anti-tumor responses. Using the CT26 murine colon cancer cell line, Dovedi et al. obtained survival rates of 66% and 80% with fractionated RT (10 Gy in 5 fractions) combined with either a PD-1 or PD-L1 inhibitor, respectively. This synergistic response was dependent on the sequencing of therapies. Improvement in OS was only observed when anti-PD-L1 therapy was given concurrently, starting either on day 1 or 5, with fractionated RT as opposed to adjuvantly, 7 days after the completion of RT. Since fractionated RT can induce PD-1 expression in tumor infiltrating CD4^+^ and CD8^+^ T cells hours after treatment, checkpoint blockade administered at this time likely blocks the PD-1/L1 signaling axis thereby augmenting T cell responses and preventing T cell anergy (67).

The efficacy of immunotherapy is also affected by radiation dose fractionation. Using the TSA mouse breast cancer model, Dewan et al. implanted tumors at two separate sites. Established tumor at one site was treated with either 20 Gy in a single fraction, 24 Gy in 3 fractions, or 30 Gy in 5 fractions with or without the addition of 9H10. Combination therapy with fractionated radiation, but not single fraction RT, resulted in almost complete tumor regression and significantly delayed growth in the non-irradiated tumor. Interestingly, 24 Gy in 3 fractions was significantly more effective than 30 Gy in 5 fractions at inhibiting tumor growth and generating tumor specific CD8^+^ CTL responses (73, 74).

Taken together, the preclinical data suggests that 14–24 Gy delivered in 2–3 fractions with concurrent ICI may be the optimal dose and fractionation of radiation, and sequencing of therapies for generating robust anti-tumor CTL responses. Whether this is true in humans as well remains to be elucidated.

## The Potential Importance of Field Size and Elective Nodal Irradiation

Another consideration for the radiation-oncologist, in addition to dose and fractionation, is determination of targets and field size. To aid with target delineation, the international commission on radiation units (ICRU) developed the concept of gross target volume (GTV), clinical target volume (CTV), and planning target volume (PTV). In brief, the GTV covers all gross disease observed on physical exam and on imaging studies. The CTV encompasses the GTV plus an additional margin ranging from a few millimeters to several centimeters with the goal of covering areas of suspected subclinical disease or disease extension. The PTV is a margin added to the CTV which accounts for errors in daily patient positioning and instrument accuracy which may in turn affect target location (75). As these margins are applied volumetrically, it quickly becomes apparent that their summation leads to a field size that is substantially larger than the tumor.

From an immunologic perspective, the effects of exposing large volumes of healthy tissue to radiation remains unclear. For instance, lymphocytes that traverse through this defined margin of unaffected tissue to reach the tumor may be eradicated by radiation before they are able to illicit an effective anti-tumor response. Injury to the healthy neighboring tissue itself may also promote an anti-inflammatory environment through the secretion of cytokines and the upregulation of immunosuppressive markers such as PD-L1 in an attempt to protect itself from immune mediated killing, thus stifling immune responses even further. SRS and SBRT, techniques which often limit margin sizes to only a few millimeters, may be one way to mitigate these potential complications while preserving the tumoricidal and immune stimulating effects of radiation and is an ongoing area of investigation.

In an attempt to prevent regional disease recurrence, radiation-oncologists will often treat DLN regions that are at high risk for disease based on findings from historical surgical series and analysis of recurrence patterns. This technique is termed elective nodal irradiation (ENI) and when employed, is considered part of the CTV. Given the extensive lymphatic drainage of the head and neck, ENI is commonly used when treating in either the adjuvant or definitive setting despite a surgically negative or clinically negative neck, respectively. The DLN however are one of the major locations where DC priming of CTLs occurs and is therefore essential in generating tumor specific CD8^+^ T cell responses. In fact, Sharabi et al. demonstrated that the DLN are the primary site for the cross-presentation of MHC class I tumor antigens seen after stereotactic radiation and can be enhanced by either anti-PD-1 therapy or ablation of Tregs (76). Thus, surgical ablation and ENI may actually curtail the efficacy of immunological responses. In fact, Takeshima et al. demonstrated that the generation of tetramer positive tumor specific CTL were significantly reduced after radiation in mice whose DLN were either surgical removed or genetically defective compared with mice whose DLN were intact (77). Recently, Marciscano et al. demonstrated that mice that underwent irradiation of both the tumor and DLNs experienced a statistically significant reduction in the number of intratumoral antigen specific CD8^+^ effector T cells compared with those receiving irradiation of the tumor alone. This was in part mediated by a decrease in chemokine expression [C-C Motif Chemokine Ligand 5 (CCL5), CXCL10, and CCL3]. Survival was significantly worse in animals receiving radiation to the tumor and DLN compared with those receiving RT to the tumor alone when treated with concurrent immune checkpoint blockade (78). Thus, taken together, these pre-clinical studies suggest that perhaps avoiding both the surgical removal and irradiation of DLN may be necessary to maximize the immunogenic response to combined radiation and immunotherapy. Whether this is true in humans however has yet to be ascertained.

While the immune stimulating potential of these techniques are intriguing, it is important for the reader to bear mind that changes in dose, reduction of margins, and omission of elective lymph node irradiation may come at the cost of local tumor control and thus goes against the current standard of care. These factors however warrant additional investigation and should be considered as evaluable metrics in future clinical trials.

## Clinical Trials Evaluating Immune Modulation in HNSCC

While HNSCCs are most commonly caused by either viral infection (HPV, EBV), tobacco use, and/or alcohol consumption, its progression is closely linked to immune escape. Thus, it stands to reason that mechanisms such as immune checkpoint blockade, which are aimed at overcoming self-tolerance and reengaging the immune system, may lead to tumor eradication and improved long term control. This strategy has already shown promise in clinical trials outside of the head and neck area, (79–81), and as PD-L1 is expressed in anywhere from 46 to 100% of cases depending on cut off for positivity and detection technique, the use of anti-PD-1/L1 therapy also has a biological basis in HNSCCs (82, 83).

Nivolumab, a human IgG4 monoclonal antibody against PD-1, was tested in a phase III open-label clinical trial (CheckMate 141) in 361 patients with recurrent or metastatic HNSCC who experienced disease progression within 6 months of receiving platinum-based chemotherapy. Patients were randomized to receive either nivolumab (at a dose of 3 mg per kilogram of body weight every 2 weeks) or investigator's choice single-agent standard therapy consisting of either methotrexate, docetaxel, or cetuximab. OS was significantly improved in the nivolumab arm: median OS was 7.5 months [95% confidence interval (CI), 5.5 to 9.1] with nivolumab vs. 5.1 months (95% CI, 4.0 to 6.0) with standard therapy. The rate of grade 3 and 4 adverse events was significantly lower with nivolumab (13.1%) compared with the standard arm (35.1%), without deterioration of patient reported quality of life scores (4).

KEYNOTE-040 was a similar open-label, phase III clinical trial including 495 patients with recurrent or metastatic HNSCC after a platinum-based chemotherapy which used pembrolizumab, another PD-1 monoclonal antibody. Patients were randomized to either monotherapy with pembrolizumab or standard of care chemotherapy. While the final publication is still pending at the time of this writing, the results were initially presented at the European Society of Medical Oncology meeting in 2017. Despite a 19% improvement in OS compared with standard of care therapy, the study failed at that time to reach its primary endpoint which was pre-specified to detect significance with a hazard ratio of 0.80. However, patients with PD-L1 expression levels >50% had significant improvement in OS with the use of pembrolizumab vs. standard chemotherapy, 11.6 vs. 7.9 months, respectively (HR = 0.54; 95% CI = 0.35 to 0.82, *p* = 0.0017). As additional survival data for this patient population was collected, updated information using the same data cutoff date was presented at the American Association for Cancer Research Annual Meeting in 2018. With the more complete dataset, the HR for OS now reached 0.8 (*p* = 0.0161), reinforcing the utility of pembrolizumab for platinum-refractory recurrent or metastatic HNSCC (84).

While nivolumab and pembrolizumab target the PD-1 receptor, durvalumab targets the PD-1 ligand (PD-L1). In an open-label phase I/II multicenter trial, durvalumab was tested in multiple solid tumor subtypes including HNSCC. Specifically, 62 patients with recurrent or metastatic disease were treated with durvalumab at 10 mg/kg every 2 weeks for 12 months. Overall response rate was 12% and as high as 25% in patients with PD-L1 positivity. Again, ICI was well-tolerated, with Grade 3 or higher toxicity being reported in only 7% of patients (85). The HAWK study, an international phase II trial evaluating the objective response rates of durvalumab in 111 immunotherapy-naïve patients with platinum refractory recurrent/metastatic HNSCC with ≥25% PD-L1 expression, revealed a response rate of 16.2% in HPV positive patients and 10.9% in HPV negative patients. PFS and OS were 2.1 and 7.1 months, respectively. Adverse events of any grade was 57.1 and 8% for greater than grade 3 toxicity (86). Lastly, a phase II randomized trial in recurrent or metastatic patients with PD-L1 low or negative tumors (< 25% expression on tumor cells) known as CONDOR failed to demonstrate enhanced efficacy of adding the CTLA-4 antibody tremelimumab to single agent durvalumab (ORR 7.8% vs. 9.2% for combination therapy and monotherapy, respectively) (87).

The successes of ICI therapy in the second line metastatic and recurrent setting has spurred significant interest in the use of PD-1 and PD-L1 ICIs in the first line for recurrent and/or metastatic disease as well as in locally advanced disease. Recently, the results from KEYNOTE 048, a 3-arm phase III trial using either pembrolizumab monotherapy, pembrolizumab in combination with platinum and 5-FU, or standard of care platinum and 5-FU plus cetuximab (“EXTREME” regimen) in the first-line treatment of recurrent or metastatic HNSCC, were presented. The primary endpoints included OS and PFS in all patients as well as in patients with positive PD-L1 expression as defined by a combined positive score (CPS), which includes the total number of PD-L1 stained cells (tumor cells, lymphocytes, macrophages) divided by the total number of viable tumor cells in a field multiplied by 100. For CPS ≥20%, patients treated with pembrolizumab had a median OS of 14.9 months vs. 10.7 months for patients treated with the EXTREME regimen (*p* = 0.00007). In all patients, regardless of CPS, when pembrolizumab was added to a chemotherapy backbone of platinum and 5-fluorouracil patients had longer OS than if they were treated with EXTREME (median OS 13.0 months vs. 10.7 months, *p* = 0.0034). Additional analyses including the efficacy of these treatments in CPS < 1 patients, the use of second-line treatments in each arm, and the impact of HPV have not yet been reported (88).

### Combining Radiation and Immunotherapy in the Clinic

As discussed above, pre-clinical studies clearly demonstrate that radiation modulates the immune system in ways that, when combined with immunotherapy, has the potential to augment treatment responses. In the clinic, this has been demonstrated through development of what is known as the abscopal (“ab” -away from, “scopus”- target) response. First coined by R H mole in 1953, it describes a phenomenon that can occur when localized radiation therapy induces regression of disease at a distant site. In 2012, Michael Postow published a case report of a patient with metastatic melanoma who demonstrated disease progression after being on treatment with ipilimumab for over a year. She subsequently underwent a course of palliative SBRT, 28.5 Gy in 3 fractions, to a single painful paraspinal lesion, followed by an additional dose of ipilimumab 1 month later. Post treatment imaging at 3 months revealed regression of the irradiated lesion as well as the non-irradiated areas of disease in the hilum and spleen. This corresponded to increased antibody titers for NY-ESO-1, an antigen frequently expressed by melanoma, as well as an increase in effector CD4^+^ T cells (8). Together these findings suggest that radiation triggered antigen release that with the addition of ipilimumab was able to generate a systemic immune response.

The data on efficacy of combined therapy in the clinical setting is still lacking while many trials are underway. A small retrospective study assessed treatment outcomes of 37 patients on immunotherapy (nivolumab 83.8%, atezolizumab 10.8%, pembrolizumab 5.4%) with brain metastases receiving SRS to a total of 85 lesions. They demonstrated that patients treated with concurrent SRS and ICI had longer OS and reduced rates of distant brain failure (DBF) than those who received SRS either before or after starting ICI (1 year OS, 87.3% vs. 70.0% vs. 0%, *p* = 0.008; 1 year DBF, 38.5% vs. 65.8% vs. 100%, *p* = 0.042). Additionally, local control was significantly improved with combination therapy at 1 year (100% vs. 72.3%, *p* = 0.016) (89).

Despite the excitement generated by this report, concerns about the possibility of increased toxicity with combined therapy exist (90). For instance, a recent retrospective review from the Dana Farber Cancer Institute examined 480 cases of patients with newly diagnosed brain metastases treated with SRS, 115 of whom were also on treatment with checkpoint inhibitors (ipilimumab, pembrolizumab, or nivolumab). Patients who received ICI were 2.5 times more likely to develop radionecrosis; the highest risk (HR 4.7) was in patients with melanoma receiving ipilimumab (91).

### Combining Radiation and Immunotherapy in Head and Neck Cancer

With regards to HNSCC, the majority of available clinical data currently focuses on the safety of combining ICI and radiation. Preliminary toxicity results have been published from GORTEC 2015-01 (“PembroRad”). This phase II trial randomized patients with locally advanced head and neck squamous cell carcinoma (LA-HNSCC) who were unfit to receive cisplatin to either RT with cetuximab or RT with pembrolizumab. Of the 133 accrued patients, 92% completed at least 33 fractions of RT and 87% received 3 courses of ICI. While rates of Grade 3 dermatitis, rash, and mucositis were significantly reduced in the pembrolizumab arm, rates of dysthyroidism were significantly increased compared to those treated with cetuximab. In this study it was somewhat concerning that treatment-related mortality was higher than previous GORTEC studies in both arms, possibly reflecting patient selection (i.e., the inclusion of high risk patients due to age and/or comorbidities that made them cisplatin-ineligible) (92). Efficacy results of the PembroRad trial are still pending.

A smaller phase 2 trial evaluated the safety and efficacy of durvalumab with concurrent palliative RT in 10 patients with either inoperable or metastatic disease with a minimum of 5% PD-L1 expression across multiple disease sites. Five patients reported radiation related adverse events of Grade 1 or 2 severity, and no one experienced grade 3 or greater toxicity. The most common side effect was mucositis which was transient and resolved in <1 week (93).

Other trials have evaluated the combination of ICI with radiotherapy and cisplatin in locally advanced HNSCC. Overall there have been no safety concerns with this approach. Specifically, Powell et al. presented the results of a phase I clinical trial investigating the role of pembrolizumab with cisplatin based chemo-radiation for LA-HNSCC at the national meeting of the American Society of Clinical Oncology (ASCO) (94). Of the 27 patients with AJCC 7th edition stage III or IV oropharyngeal, hypopharyngeal, and laryngeal squamous cell carcinomas, 78% of patients completed all planned doses of ICI while 3 patients discontinued treatment due to either Grade 2 peripheral neuropathy, Grade 1 Lhermitte syndrome, or Grade 3 elevation in liver transaminases. All patients successfully completed radiation to the planned dose of 70 Gy without significant delay, defined as >5 days, and 85% received the target dose of cisplatin. One patient died due to a concurrent illness unrelated to the treatment regimen.

Similarly, the combination of nivolumab with cisplatin in either 3 weekly or weekly dosing was shown to be safe without unexpected toxicities (95). In RTOG 3504 pilot trial, patients with newly diagnosed HNSCC who were considered either intermediate risk (p16+, oropharynx T1-2N2b-N3/T3-4N0-3, >10 pack-years smoking; or T4N0-N3, T1-3N3, ≤10 pack-years) or high-risk (oral cavity, larynx, hypopharynx, or p16- oropharynx, stage T1-2N2a-N3 or T3-4N0-3) were enrolled and treated with nivolumab in addition to cisplatin and radiation. Cisplatin was given at either a low weekly dose (40 mg/m^2^) or high dose (100 mg/m^2^ every 3 weeks). Nivolumab was given at a dose of 240 mg every 14 days when in conjunction with the weekly dose cisplatin and as a single dose of 240 mg followed by 360 mg every 21 days with the high dose cisplatin. After the conclusion of concurrent chemoradiotherapy, patients were planned to continue on 480 mg every 28 days for 7 doses. As above, all patients were able to complete the prescribed dose of radiation therapy, 70 Gy in 35 fractions. Of the 17 patients available for analysis at the time interim data was presented, 15 were able to receive at least 70% of their planned platinum dose. Three patients discontinued cisplatin, 2 for an allergic reaction and 1 for cholecystitis. Three patients also discontinued nivolumab for known side-effects related to the drug. One grade 4 AE of elevated amylase was reported but resolved. This trial demonstrated the safety of the combination of nivolumab with chemoradiotherapy as well as the feasibility of adjuvant nivolumab after CRT.

More recently, Wise-Draper et al. reported results from a phase II trial investigating the role of neoadjuvant pembrolizumab followed by surgery and then adjuvant concurrent pembrolizumab-RT or pembrolizumab-cisplatin-RT in patients with LA-HNSCC (96). At interim analysis 16 out of 16 patients in the pembrolizumab-RT arm had no Grade 4 toxicity or delay in care due to dose-limiting toxicity, leading the authors to conclude the combined regimen is safe. The pembrolizumab-cisplatin-RT arm also had no grade 4 events reported in the 19 patients included in their preliminary data.

In order to assess the efficacy of combining ICI with SBRT in metastatic HNSCC, a phase II trial enrolled 56 patient to receive either nivolumab alone (*n* = 28) or nivolumab given with SBRT given as 9 Gy × 3 to a single lesion between the first and second doses of nivolumab (*n* = 28). Non-irradiated index lesions were followed for response. As above, the rates of grade 3 or greater treatment-related toxicities were low, occurring in 14.3% of the nivolumab alone arm and 10.7% of the nivolumab and SBRT arm. The ORR was not significantly different, 30.8% vs. 25.9%, *p* = 0.93, nor were the mPFS (1.9 months vs. 2.4 months, *p* = 0.89) or 1 year OS rates (46% vs. 54%, *p* = 0.46). Thus, they failed to demonstrate an abscopal response in the index lesions. However, subgroup analysis revealed that tumors with a high mutational burden had significantly more responders and that mutational burden predicted response regardless of viral status (97).

Taken together these studies suggest that ICI can be safely administered concurrently with radiation therapy without exacerbation of expected toxicities. Most importantly, however, they highlight the need for additional prospective data looking at efficacy. Fortunately, in addition to the aforementioned trials whose efficacy results are still pending, there are over 40 phase I to III clinical trials aimed at addressing exactly this question in head and neck cancers alone (Table 1).

**Table 1:** Clinical trials incorporating checkpoint inhibitors and radiation therapy in head and neck squamous cell carcinoma.

**NCT ID#**	**Phase**	**Title**	**ICI**	**Treatment arms**
**PHASE 1**				
NCT03539198	NA	A prospective observational study of study of proton SBRT and immunotherapy for recurrent/progressive locoregional or metastatic head and neck cancer	Nivolumab	Loading dose of Nivolumab on D-14 then concurrently w/RT; Proton SBRT 5 fxs; 35–45 Gy)
NCT02764593	1	Safety Testing of Adding Nivolumab to Chemotherapy in Patients With Intermediate and High-Risk Local-Regionally Advanced Head and Neck Cancer	Nivolumab	Loading dose on D-14 then concurrently w/cisplatin or cetuximab and RT, followed by adjuvant ICI; 70 Gy in 35 fxs; IMRT
NCT03402737	1	SBRT + Immunomodulating Systemic Therapy for Inoperable, Recurrent Head and Neck Cancer	Nivolumab	Concurrently w/RT; 6–8 Gy times 2 fxs 6–8 Gy times 3 fxs 6–10 Gy times 3 fxs 6–12 Gy times 3 fxs
NCT02318771	1	Radiation therapy and MK-3475 for patients with recurrent/metastatic head and neck cancer, renal cell cancer, melanoma, and lung cancer	Pembrolizumab	Arm A: Adjuvant (3–17 days post RT); Arm B: Concurrent; A1 and B1: 8 Gy in 1 fx Arms A2 and B2: 20 Gy in 5 fxs
NCT02586207	1	Pembrolizumab in combination with CRT for LA-SCCHN	Pembrolizumab	Loading dose on D-7 then concurrent q3 weeks with cisplatin-RT;70 Gy in 35 fxs
NCT02819752	1	Pembrolizumab combined with chemoradiotherapy in squamous cell carcinoma of the head and neck (PEACH)	Pembrolizumab	Concurrently with CRT; Standard therapy
NCT03509012	1	Immunotherapy in combination with chemoradiation in patients with advanced solid tumors (CLOVER)	Durvalumab	Various regimens
NCT02938273	1	Bioimmunoradiotherapy (Cetuximab/RT/Avelumab)	Avelumab	Loading dose D-7 then concurrently w/cetuximab-RT; 70 Gy over 7 weeks
NCT01935921	1	Ipilimumab, cetuximab, and intensity-modulated radiation therapy in treating patients with previously untreated stage III-IVB head and neck cancer	Ipilimumab	Concurrently w/cetuximab-RT; IMRT daily for 7 weeks
NCT01860430	1	A phase Ib trial of concurrent cetuximab (ERBITUX®) and intensity modulated radiotherapy (IMRT) With ipilimumab (YERVOY®) in locally advanced head and neck cancer	Ipilimumab	Concurrently w/cetuximab-RT; 70–74 Gy in 2 Gy daily fxs; IMRT
NCT03162731	1	Nivolumab, ipilimumab, and radiation therapy in treating patients with stage IVA-B head and neck cancer	Nivolumab, Ipilimumab	Loading dose of Nivolumab on D-21 then concurrently w/RT and ipilimumab; 70 Gy in 35 fxs
NCT03529422	1	Durvalumab and Tremelimumab with radiotherapy for adjuvant treatment of intermediate risk SCCHN	Durvalumab, Tremelimumab	Concurrently w/RT; 60 Gy in 30 fxs; IMRT
NCT03317327	1/2	REirradiation and programmed cell death protein 1 (PD-1) blockade on recurrent squamous cell head and neck tumors (REPORT)	Nivolumab	Concurrently w/RT; 60 Gy in 1.5 Gy fxs BID for 4 weeks
NCT03247712	1/2	Neoadjuvant immunoradiotherapy in head and neck cancer	Nivolumab	Concurrently w/RT; Arm 1: 8 Gy times 5 fxs daily Arm 2: 8 Gy times 3 fxs QOD
NCT02759575	1/2	A study of chemoradiation plus pembrolizumab for locally advanced laryngeal squamous cell carcinoma	Pembrolizumab	Loading dose on D-21 then concurrently w/cis-RT; 70 Gy in 35 fxs
NCT03114280	1/2	Pembrolizumab and induction chemotherapy in head and neck squamous cell carcinoma (PICH study) (PICH)	Pembrolizumab	Neoadjuvant with chemotherapy, followed by concurrent chemoradiotherapy with carboplatin; Unknown dose or RT
NCT03051906	1/2	Durvalumab, cetuximab, and radiotherapy in head neck cancer (DUCRO-HN)	Durvalumab	Concurrently w/cetuximab-RT followed by adjuvant therapy; 69.96 Gy in 2.12 Gy fxs
NCT03212469	1/2	A trial of durvalumab and tremelimumab in combination with SBRT in patients with metastatic cancer (ABBIMUNE)	Durvalumab, tremelimumab	SBRT
NCT03283605	1/2	Immunotherapy and SBRT for metastatic head and neck carcinomas	Durvalumab, tremelimumab	Neoadjuvant then concurrently w/RT; SBRT
NCT03522584	1/2	Durvalumab, tremelimumab, and stereotactic body radiation therapy in treating participants with recurrent or metastatic head and neck squamous cell carcinoma	Durvalumab, tremelimumab	Loading dose D-14 then concurrently w/RT; SBRT QOD
**PHASE 2**				
NCT02684253	2	Screening trial of nivolumab with image guided, stereotactic body radiotherapy (SBRT) vs. nivolumab alone in patients with metastatic head and neck squamous cell carcinoma (HNSCC)	Nivolumab	Concurrently w/RT; SBRT 27 Gy in 3 fxs QOD
NCT03521570	2	Intensity-modulated radiation therapy and nivolumab for recurrent or second primary head and neck squamous cell cancer	Nivolumab	Loading dose of Nivolumab on D-14 then concurrently with RT; IMRT daily for 6–6.5 weeks
NCT03107182	2	Chemotherapy and locoregional therapy trial (surgery or radiation) for patients with head and neck cancer (OPTIMA-II)	Nivolumab	Induction with chemotherapy followed by adjuvant therapy; Dose de-escalated to 45–50 Gy (Arm 2 and 3) or conventional dose to 70 Gy (Arm 4)
NCT03511391	2	Checkpoint inhibition in combination with an immunoboost of external body radiotherapy in solid tumors (CHEERS)	Pembrolizumab, Nivolumab	Concurrently with RT; SBRT 8 Gy times 3 fxs
NCT03313804	2	Priming immunotherapy in advanced disease with radiation	Pembrolizumab, Nivolumab, Atezolizumab	Concurrently w/RT; SBRT with BED >100 Gy or 30 Gy in 3 Gy fxs
NCT02641093	2	Phase II trial of adjuvant cisplatin and radiation with pembrolizumab in resected head and neck squamous cell carcinoma	Pembrolizumab	Loading dose 1 week prior to surgery then concurrently w/ cis-RT; 60–66 Gy in 2 Gy fxs
NCT02707588	2	Tolerance and efficacy of pembrolizumab or cetuximab combined with RT in patients with locally advanced HNSCC (PembroRad)	Pembrolizumab	Concurrently w/RT; 69.96 Gy in 2.12 Gy daily fxs
NCT02609503	2	Pembrolizumab + radiation for locally Adv SCC of the Head and Neck (SCCHN) Not eligible cisplatin	Pembrolizumab	Concurrently w/RT; IMRT daily for 7 weeks
NCT02296684	2	Immunotherapy with MK-3475 in surgically resectable head and neck squamous cell carcinoma	Pembrolizumab	Arm 1: Neoadjuvant and adjuvant therapy Arm 2: Neoadjuvant;
NCT02289209	2	Reirradiation With pembrolizumab in locoregional inoperable recurrence or second primary squamous cell CA of the head and neck	Pembrolizumab	Concurrently w/RT; 1.2 Gy BID for 5 days a week for 5 weeks
NCT02777385	2	Pembrolizumab in combination with cisplatin and intensity modulated radiotherapy (IMRT) in head and neck cancer	Pembrolizumab	Arm 1: adjuvant 3 weeks post cisplatin-RT Arm 2: concurrently with cisplatin-RT; 70 Gy in 35 fxs; IMRT
NCT03085719	2	Targeting PD-1 therapy resistance with focused high or high and low dose radiation in SCCHN	Pembrolizumab	Concurrently w/RT; High (3 fxs) vs. low dose (2 fxs)
NCT03532737	2	Concomitant immune check point inhibitor with radiochemotherapy in head and neck cancer	Pembrolizumab	Loading dose on D-14 then concurrently w/either cetuximab or cis-RT; 66–70 Gy in 30–35 fxs; IMRT
NCT03057613	2	The addition of pembrolizumab to postoperative radiotherapy in cutaneous squamous cell cancer of the head and neck	Pembrolizumab	Concurrently w/and adjuvantly to post-op RT; 60–66 Gy for 6 weeks; IMRT
NCT03383094	2	Chemoradiation vs. immunotherapy and radiation for head and neck cancer	Pembrolizumab	Concurrently w/and adjuvant to cis-RT; 70 Gy in 33–35 fxs
NCT03546582	2	SBRT +/– pembrolizumab in patients with local-regionally recurrent or second primary head and neck carcinoma (KEYSTROKE)	Pembrolizumab	Adjuvant to RT; SBRT
NCT03386357	2	Radiotherapy with pembrolizumab in metastatic HNSCC	Pembrolizumab	Concurrently w/RT; 12 Gy times 3 fxs
NCT03624231	2	Feasibility and efficacy of Durvalumab+Tremelimumab+RT and Durvalumab+RT in Non-resect. Locally advanced HPVnegativ HNSCC (DURTRE-RAD)	Durvalumab, Tremelimumab	Loading dose D-14 then concurrently w/ RT, followed by adjuvant therapy; 70 Gy in 35 fxs over 7 weeks
NCT03426657	2	Radiotherapy with double checkpoint blockade of locally advanced HNSCC	Durvalumab, Tremelimumab	Concurrently w/RT followed by durva monotherapy; 70 Gy in 35 fxs
NCT03258554	2/3	Radiation therapy with Durvalumab or Cetuximab in treating patients with stage III-IVB head and neck cancer who cannot take cisplatin	Durvalumab	Loading dose D-14 then concurrently w/RT; IMRT
**PHASE 3**				
NCT03349710 (closed to slow accrual)	3	Nivolumab or nivolumab plus cisplatin, in combination WITH radiotherapy in patients with cisplatin-ineligible or eligible locally advanced squamous cell head and neck cancer	Nivolumab	RT w/cis and nivo vs. RT w/cis RT w/cetuximab vs. RT w/nivo 70 Gy in 35 fractions over 7 weeks; IMRT
NCT03576417	3	A trial evaluating the addition of nivolumab to cisplatin-rt for treatment of cancers of the head and neck (NIVOPOSTOP)	Nivolumab	Loading dose of Nivolumab on D-21 then concurrently w/cis-RT; 66 Gy over 6.5 weeks; IMRT
NCT03040999	3	Study of pembrolizumab (MK-3475) or placebo with chemoradiation in participants with locally advanced head and neck squamous cell carcinoma (MK-3475-412/KEYNOTE-412)	Pembrolizumab	Loading dose then concurrently w/cis-RT; 70 Gy in 35 fxs over either 6 (accelerated) or 7 (standard) weeks
NCT02952586	3	Study to compare avelumab in combination with standard of care chemoradiotherapy (SoC CRT) vs. SoC CRT for definitive treatment in patients with locally advanced squamous cell carcinoma of the head and neck (Javelin head and neck 100)	Avelumab	Concurrently w/cisplatin-RT; 70 Gy in 35 fxs; IMRT
NCT02999087	3	Randomized trial of avelumab-cetuximab-radiotherapy vs. SOCs in LA SCCHN (REACH)	Avelumab	Concurrently w/cetuximab-RT; 69.96 Gy in 2.12 Gy daily fxs; IMRT
NCT03700905	3	Study of nivolumab alone or in combination with ipilimumab as immunotherapy vs. standard follow-up in surgical resectable HNSCC after adjuvant therapy (IMSTAR-HN)	Nivolumab Ipilimumab	Neoadjuvant Nivolumab followed by surgery, adjuvant cisplatin-RT (66 Gy in 33 fx), and adjuvant Ipilimumab and Nivolumab
NCT03673735	3	Maintenance immune check-point inhibitor following post-operative chemo-radiation in subjects with hpv-negative HNSCC (ADHERE)	Durvalumab	Induction Durvalumab followed by cisplatin-RT (66 Gy in 33 fx), and maintenance Durvalumab
NCT03258554	3	Radiation therapy with durvalumab or cetuximab in treating patients with stage III-IVB head and neck cancer who cannot take cisplatin	Durvalumab	Concurrently with RT (IMRT)

*Selected clinical trials incorporating the use of one or more immune checkpoint inhibitor and radiation therapy are included below. When available, the dosing and sequencing for the trials is included. SBRT, Stereotactic Body Radiotherapy; RT, radiation therapy; fxs, fractions; Gy, gray; ICI, immune checkpoint inhibitor; CRT, chemoradiotherapy; IMRT, intensity-modulated radiation therapy; LA-SSCHN, locally advanced squamous cell carcinoma of the head and neck; BED, biologically equivalent dose*.

## Challenges and Future Directions

It is now evident that radiation, through a plethora of diverse mechanisms, has the ability to generate anti-tumor immune responses which can be potentiated by immune checkpoint inhibition. Despite the progress made over the last few years in our understanding of this response, numerous questions remain. It is unclear as to how ICI should be delivered with RT i.e., neoadjuvant, concurrent, adjuvant, or in some combination of the three. Furthermore, it remains to be seen whether combining anti-CTLA4 and anti-PD-1/L1 therapy, given their non-redundant nature, truly improves responses or whether the toxicity precludes the use of such regimens. Both of these questions are currently being addressed in numerous clinical trials (from phase I to phase III) in HNSCC that are listed in Table 1.

For the radiation oncologist, there are also the questions of total dose, fraction size, inter-fraction time, target selection, and field size. Preclinical data appears to support the use of large doses in few fractions in producing optimal immune responses, but this still requires validation in humans. In terms of target, radiation oncologists typically select symptomatic lesions where RT may provide palliative relief. This however may not be the best methodology as it is unknown whether targeting bone vs. soft tissue, or even those located in so-called “sanctuary sites” such as the CNS, may confer better outcomes. Lastly, with improvements in targeting it is unclear what field sizes would improve responses. For instance, if tighter tumor margins reduce unwanted eradication of trafficking CTLs or if larger margins increase antigen exposure allowing for improved DC uptake and CTL priming. Therefore, in order to truly maximize the potential of these therapies, more research in both the preclinical and clinical setting is warranted.

## Author Contributions

GM produced the original draft of the article with guidance and editing from VB-A, BL, AA, and JJ.

### Conflict of Interest Statement

AA receives research support from Bristol Myers Squibb, and provides paid consultative services for Bristol Myers Squibb, Merck Serono, Aspyrian, and Debiopharm. JJ receives research support from Bristol Myers Squibb, Astra Zeneca, and Merck. The remaining authors declare that the research was conducted in the absence of any commercial or financial relationships that could be construed as a potential conflict of interest.
